# How two extraembryonic epithelia became one: serosa and amnion features and functions of *Drosophila*'s amnioserosa

**DOI:** 10.1098/rstb.2021.0265

**Published:** 2022-12-05

**Authors:** Urs Schmidt-Ott, Chun Wai Kwan

**Affiliations:** ^1^ Department of Organismal Biology and Anatomy, University of Chicago, 1027 East 57th Street, Chicago, IL 60637, USA; ^2^ Laboratory for Epithelial Morphogenesis, RIKEN Center for Biosystems Dynamics Research, 2-2-3 Minatojima-minamimachi, Chuo-ku, Kobe, Hyogo 650-0047, Japan

**Keywords:** evolutionary developmental biology (evo-devo), morphogenesis, dorsal closure, germ band retraction, Diptera

## Abstract

The conservation of gene networks that specify and differentiate distinct tissues has long been a subject of great interest to evolutionary developmental biologists, but the question of how pre-existing tissue-specific developmental trajectories merge is rarely asked. During the radiation of flies, two extraembryonic epithelia, known as serosa and amnion, evolved into one, called amnioserosa. This unique extraembryonic epithelium is found in fly species of the group Schizophora, including the genetic model organism *Drosophila melanogaster*, and has been studied in depth. Close relatives of this group develop a serosa and a rudimentary amnion. The scuttle fly *Megaselia abdita* has emerged as an excellent model organism to study this extraembryonic tissue organization. In this review, development and functions of the extraembryonic tissue complements of *Drosophila* and *Megaselia* are compared. It is concluded that the amnioserosa combines cells, genetic pathway components and functions that were previously associated either with serosa development or amnion development. The composite developmental trajectory of the amnioserosa raises the question of whether merging tissue-specific gene networks is a common evolutionary process.

This article is part of the theme issue ‘Extraembryonic tissues: exploring concepts, definitions and functions across the animal kingdom’.

## 1. Introduction

Most insect embryos develop with the help of support epithelia, called serosa and amnion [1,2]. In flies (Diptera) [3], these epithelia have merged [4,5] and offer a very instructive model for how tissue-specific gene networks and morphogenetic processes can be combined in the course of evolution.

**Figure 1.**
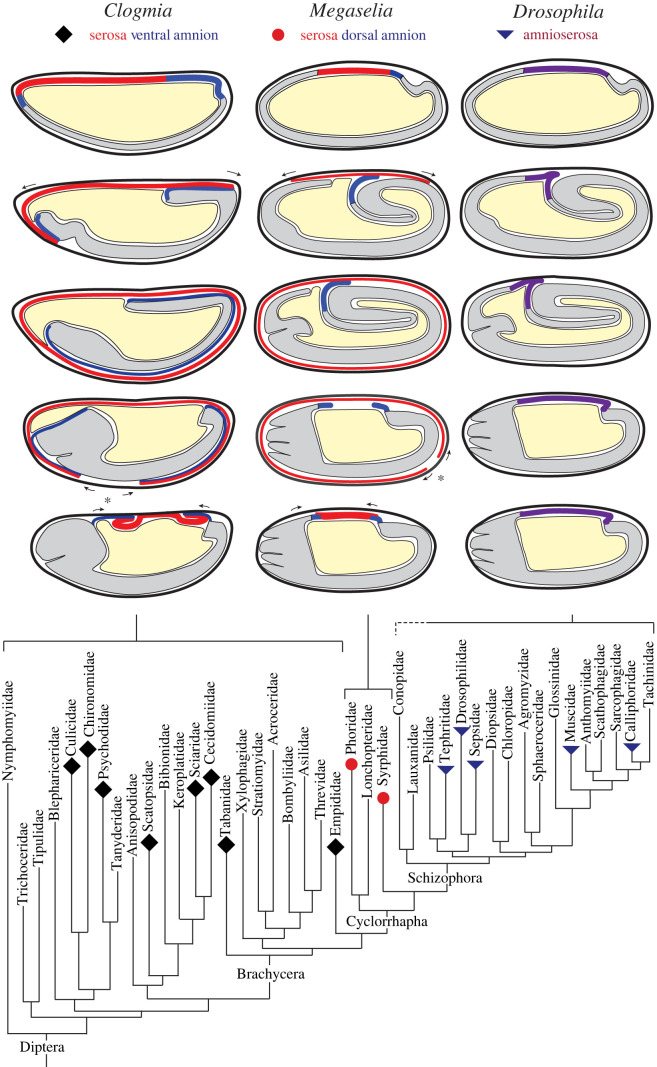
Sketches of three modes of extraembryonic development and their phylogenetic occurrence in Diptera. The eggshell is depicted as a black outer line, the serosa in red, the amnion in blue, the amnioserosa in purple, the germ band in grey, and the yolk sac in light yellow. Anterior is left and dorsal up. Arrows indicate the direction of extraembryonic tissue movement. The simplified family tree of the Diptera is based on [3]. The documented occurrence of three modes of extraembryonic development is based on [5,6]. *Clogmia* is a representative of the Psychodidae, *Megaselia* of the Phoridae, and *Drosophila* of the Drosophilidae.

Dipteran embryos develop from a single cell layer (blastoderm) that encloses the central yolk sac. In lower dipterans (non-cyclorrhaphan Diptera), as in most insects, dorsal or antero-dorsal blastoderm folds and closes about the developing germ band, thereby forming an epithelium underneath the eggshell (serosa) and an epithelium that lines the outer side of the embryo epidermis (amnion) (figure 1) [7,8]. The serosa secretes a cuticle [7,9,10] and mounts a strong innate immune response when the developing embryo suffers bacterial infection [11,12]. The function of the amnion is not understood. Both extraembryonic epithelia rupture and retract into the yolk before the epidermal flanks close the embryo along the dorsal midline [8,13–17].

In the cyclorrhaphan lineage of dipterans, which includes the genetic model organism *Drosophila melanogaster*, extraembryonic tissue has been extensively modified. *Drosophila* develops a single extraembryonic epithelium, called amnioserosa [18]. Like an amnion, the amnioserosa connects to the leading edge of the dorsal epidermis but instead of lining the germ band epidermis, it covers the dorsal side of the yolk sac, thereby closing the embryo until the epidermal flanks have met at the dorsal midline above the internalized remnants of the amnioserosa.

The genetic tools that are available for *D. melanogaster* have enabled a detailed analysis of the genetic underpinnings and functions of the amnioserosa in morphogenesis [19,20]. The goal of this review is to provide phylogenetic context for these studies and to assess whether the amnioserosa combines developmental and functional traits of the serosa and the amnion as seen in the most closely related fly species that retain both tissues.

## 2. Evolution of the amnioserosa

The amnioserosa is found in schizophoran fly species [6,21,22], a large subgroup of the Cyclorrhapha that radiated in the Tertiary [3,23], while representatives of basal-branching cyclorrhaphan lineages, such as the hover fly *Episyrphus balteatus* (Syrphidae) and the scuttle fly *Megaselia abdita* (Phoridae), develop with a serosa and a vestigial amnion (figure 1) [22,24–26]. These findings suggest that the evolution of a vestigial amnion preceded the evolution of the amnioserosa and that the organization of extraembryonic tissue in lower cyclorrhaphan fly species reflects a condition that preceded the origin of the amnioserosa; hence these species are of special importance for reconstructing the evolution of the amnioserosa.

*Megaselia abdita* has become the model of choice for studying extraembryonic development in lower Cyclorrhapha because it is easy to rear and suitable for descriptive and functional embryological studies in the laboratory [4,27–31]. Moreover, the embryos of *M. abdita* closely resemble those of *D. melanogaster* in size and development (apart from the extraembryonic tissue), thereby providing a conserved framework of developmental stages for studying molecular and cellular differences in extraembryonic tissue specification and morphogenesis between these species [32].

**Figure 2.**
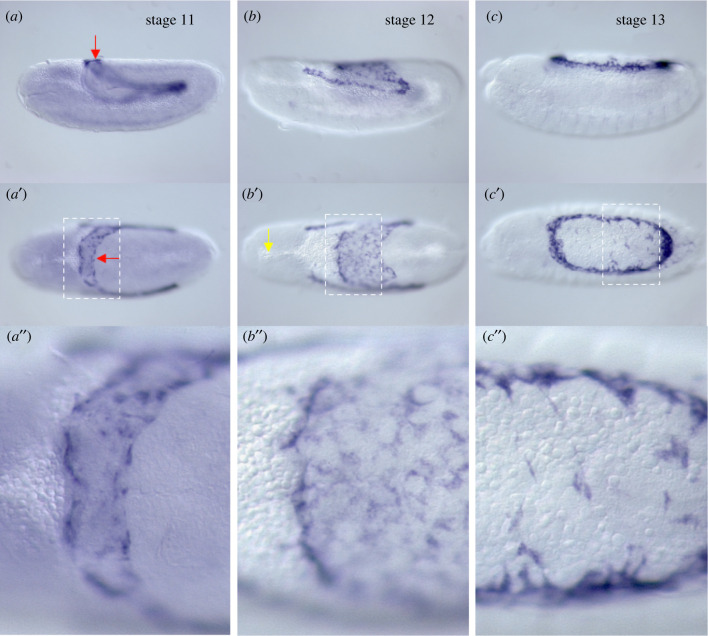
Amnion development in *Megaselia* during germ band retraction. *Megaselia* *eiger* (*egr*) RNA *in situ* hybridizations of embryos shortly before (*a*), during (*b*), and right after (*c*) germ band retraction are shown in lateral view (dorsal up) and in dorsal view. The dashed rectangles (*a*′, *b*′, *c*′) indicate enlarged regions (*a″*, *b″*, *c″*). The red arrow points to the dorsal bridge of the amnion over the tail end of the extended germ band (*a*, *a*′). Following the onset of germ band retraction, the amnion cells form cytoplasmic extensions over the yolk sac (*b*, *b′*, *b″*, *c*, *c*′, *c″*). The yellow arrow (*b*′) points to an anterior portion of the yolk sac that extends between the left and right brain anlage until the serosa ruptures and pulls back over the dorsal opening (see also figure 5). Anterior is left.

**Figure 3.**
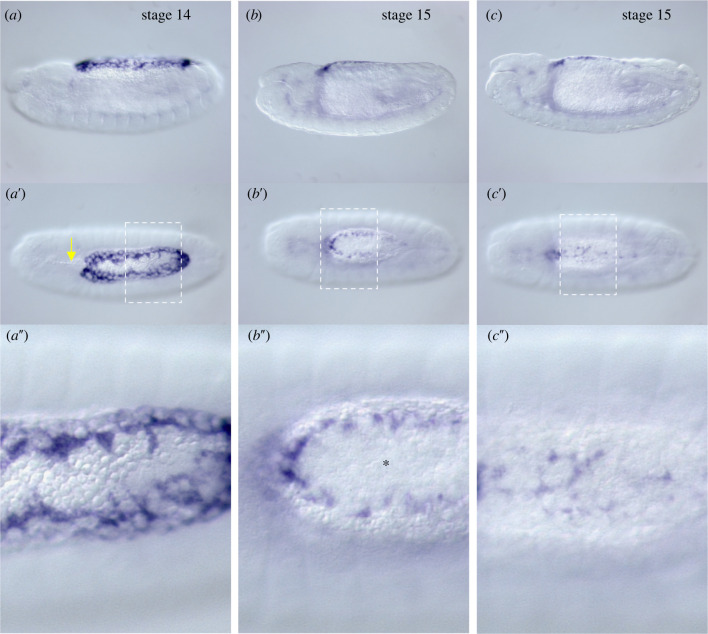
Amnion development in *Megaselia* during dorsal closure. *Megaselia* *eiger* (*egr*) RNA *in situ* hybridizations of embryos shortly before serosa rupture (*a*), after serosa rupture and contraction but before amnion closure (*b*), and after amnion closure but before zipping of the epidermis (*c*). The anterior yolk sac extension is indicated by a yellow arrow (*a*′). Note that this extension of the yolk sac retracts with the contracting serosa. The unstained contracted serosa is marked with an asterisk (*b″*). Embryos are shown in lateral view (dorsal up) and in dorsal view (*a*′, *b*′, *c*′). Dashed rectangles indicate enlarged region (*a″*, *b″*, *c″*). Anterior is left. (Online version in colour.)

In *Megaselia*, serosa cells are specified at the centre of the extraembryonic domain, which straddles the dorsal midline [6]. After gastrulation, these cells spread out underneath the eggshell and close about the extended germ band (figure 1) [22,26]. During this process, the amnion folds over the posterior germ band and—to a lesser degree—over the leading edge of the lateral epidermis [22,26]. However, once dissociated from the serosa, the amnion cells relocate to the yolk sac surface and align along the leading edge of the epidermis (figures 2 and 3) [24,33]. Using live imaging [25,26,32], it was shown that the amniotic vestige closes the embryo after the serosa ruptures and contracts towards the dorsal opening of the amnion (figure 1). The contracted serosa bends inwards and segregates into the yolk, allowing amnion cells to close over the contracted serosa along the dorsal midline [25], as described in more detail in §5. In summary, the dorsal hole in the amnion is both a consequence of serosa development and is used for the internalization of the serosa during dorsal closure.

When the specification of serosa cells is genetically suppressed or abrogated during early gastrulation by knockdown of *zerknüllt* (*zen*), a selector gene for serosal cell fate, these cells become amnion-like and form a continuous extraembryonic cell layer with the amnion, akin of an amnioserosa [22,34]. The dorsal side of these embryos is therefore closed throughout development, like in species with an amnioserosa. From a comparative perspective, it is important to note that amnioserosa development strictly correlates with the absence of a serosa epithelium underneath the eggshell and that the amnioserosa lacks characteristic features of terminally differentiated serosa cells, such as the ability to synthesize cuticle or to mount a strong innate immune response. Taken together, the developmental and comparative data raise the question of whether the amnioserosa combines cells that formerly contributed either to the serosa or to the amnion. Repurposed serosa cells could explain the correlated loss of the serosa and origin of an amnioserosa-like enlarged amnion in the course of evolution, as suggested previously [5,22]. However, a close comparison of *Drosophila* and *Megaselia* reveals serosa and amnion features in both the developmental trajectory and function of the amnioserosa.

## 3. Extraembryonic tissue specification in *Drosophila* and *Megaselia*

The amnioserosa cells of *Drosophila* are specified during blastoderm cellularization (stage 5) [35,36], downstream of a dynamic bone morphogenetic protein (BMP) signalling gradient [37–41]. During this stage, a positive feedback loop changes the activity profile of BMP signalling from a broad and shallow dorsal-to-ventral gradient to a sharp and narrow peak that determines the width of the amnioserosa anlage [42,43]. Amnioserosa specification also requires BMP-dependent expression of the homeodomain transcription factor Zerknüllt (Zen), but additional genes are required to delimit the anterior and posterior extent of this extraembryonic tissue [44,45]. Zen transforms the amnioserosa of the extending germ band into a squamous polyploid tissue in which a microtubule-dependent process elongates and reorients amnioserosa cells to promote germ band extension [20,46].

BMP signalling also activates so-called U-shaped-group genes in nested blastoderm domains [47–49]. Some of them, such as *hindsight* (*hnt)* [50] and *dorsocross (doc)* [51], function downstream of Zen, but others, such as *u-shaped* (*ush*) [47], are expressed in broader BMP-dependent domains and function in the amnioserosa as well as the adjacent epidermis, where they define epidermal competence zones that play an important role in the crosstalk between amnioserosa and dorsal epidermis [52]. When germ band extension begins to slow down (stage 9), *zen* expression is downregulated [45], and the amnioserosa is then maintained by genes of the U-shaped-group [53]. Loss-of-function mutations in U-shaped-group genes interfere with germ band retraction and dorsal closure and cause precocious disintegration of the amnioserosa. Both these morphogenetic movements require the amnioserosa [54,55].

**Figure 4.**
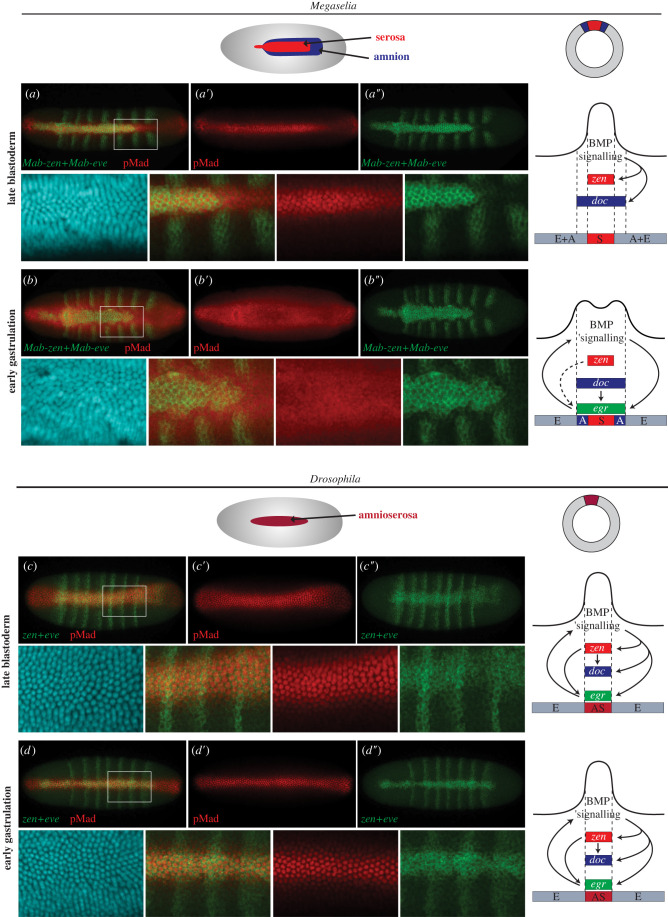
Distinct BMP signalling dynamics during extraembryonic tissue specification in *Megaselia* and *Drosophila*. *Megaselia* embryos (*a*,*b*) and *Drosophila* embryos (*c*,*d*) are shown shortly before gastrulation (stage 5) and at the beginning of gastrulation (stage 6) in dorsal view with anterior left. BMP signalling activity (pMad antibody staining) is shown in red. RNA *in situ* hybridizations with species-specific probes of *zen* and the pair-rule segmentation gene *even-skipped* (*eve*) are shown in green. Nuclear 4',6-diamidino-2-phenylindol (DAPI) staining is shown in blue. Details of the staining procedure have been reported elsewhere [33]. Schematic embryos depict the serosa (red) and amnion (blue) in *Megaselia*, and the amnioserosa (brown) in *Drosophila* at the beginning of gastrulation in dorsal view. Cross sections of these embryos and corresponding patterns of BMP-signalling and BMP-dependent gene regulatory networks are shown on the right. E, epidermis; A, amnion, S, serosa; AS, amnioserosa.

The specification of serosa and amnion in *Megaselia* also occurs downstream of BMP signalling but is achieved sequentially (figure 4) [6,24]. Serosa specification involves the formation of a sharp peak of BMP signalling during blastoderm cellularization (figure 4*a*). Amnion specification is driven by shifting the peak of BMP signalling to the rim of the germ rudiment, which gives rise to the amnion (figure 4*b*). In *Drosophila*, the sharpening of BMP gradient during blastoderm cellularization is conserved (figure 4*c*) but the broadening of the BMP gradient at the beginning of gastrulation is reduced (figure 4*d*).

Serosa specification is not only dependent on BMP signal but also on BMP-dependent Zen expression. That also applies to amnioserosa specification in *Drosophila*, although suppression of *zen* in *Drosophila* results in excess embryonic tissue rather than amnion tissue [44]. Both processes occur at a comparable stage, that is, during blastoderm cellularization. Differences in positioning the *zen*-expressing extraembryonic cells along the anterior-posterior body axis between the two species account for the fact that the *zen* domain of *Drosophila* (amnioserosa anlage) partly invaginates with prospective hindgut tissue (proctodeum) whereas the *zen* domain of *Megaselia* (serosa anlage) remains at the surface of the embryo [22]. Amnion specification is accompanied by transitioning from BMP-dependent to BMP-independent *zen* regulation so that broadening of BMP-signalling at the beginning of gastrulation does not result in a broadening of the serosa anlage. Instead, increased BMP activity at the rim of the germ rudiment suppresses embryonic pattern formation in this prospective amnion territory.

**Figure 5.**
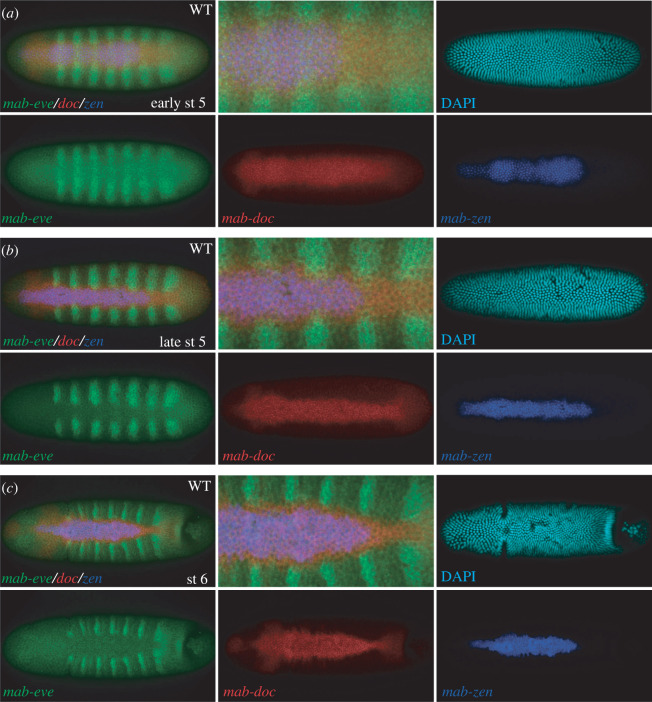
The expression of U-shaped group gene *doc* relative to *zen* and *eve* in *Megaselia*. Triple RNA *in situ* hybridizations of embryos at early and late stage 5 (*a*,*b*) and shortly after the beginning of gastrulation (stage 6) are shown in dorsal view with anterior left. *Megaselia* *doc* expression is shown in red, *Megaselia* *zen* expression in blue, and *Megaselia* *eve* in green. Nuclear DAPI stain is shown in light blue. This figure has been reproduced from [24]. (Online version in colour.)

Like the sharpening of the BMP gradient during blastoderm cellularization, the spatial changes in BMP activity at the beginning of gastrulation are driven by a positive feedback circuit [24,42]. A conserved genetic component of this feedback circuit in *Drosophila* and *Megaselia* is *eiger* (*egr*), which encodes a tumour necrosis factor-alpha ligand. However, other aspects of the feedback circuit differ between these species and may account for spatio-temporal differences in BMP signalling dynamics between the two species (figure 4). For example, the expression domains of *doc* and *hnt* form independent of *zen*, span prospective serosa and amnion tissue, function upstream of *egr*, and are part of the positive feedback loop that regulates BMP signalling (figure 5) [24].

In summary, the specification of the amnioserosa in *Drosophila* and of the serosa in *Megaselia* share similarities in when and how these tissues are specified. Both tissues are established prior to gastrulation downstream of BMP-signalling and BMP-dependent *zen* expression and develop from overlapping domains of the cellular blastoderm. However, *Drosophila*'s amnioserosa also includes cells that judged by their invagination during proctodeum formation correspond to amnion cells in *Megaselia*. In *Megaselia*, only the serosa cells express *zen*, and these cells become indistinguishable from amnion cells when Zen is knocked down before or during early gastrulation [22,34], but since Zen-deficient *Drosophila* embryos only develop embryonic tissue [44], this species seems to have lost the ability to specify amnion cells during gastrulation. Instead, *Drosophila* suppresses *zen* expression after gastrulation, thereby preventing further expansion of amnioserosa cells and their terminal differentiation as serosa cells. One may therefore ask whether *Drosophila* delays amnion specification. To address this question, we will compare the development and function of *Drosophila*'s and *Megaselia*'s extraembryonic tissue complements in post-gastrulation stages of embryonic development.

## 4. Development and function of the amnioserosa after gastrulation in *Drosophila*

The amnioserosa of *Drosophila* signals to the adjacent epidermis. In response to this unidentified signal, the dorsal-most epidermal cells (DME cells) adopt a unique cell fate [56]. These cells divide synchronously and form a ‘mitotic domain’ during the fourteens mitotic cycle [57]. This suggests that they are genetically distinct from other cell populations by the time the embryo undergoes rapid germ band extensions (stage 8). When germ band extension slows down and *zen* expression in the amnioserosa fades (stage 9) the DME cells activate Jun N-terminal kinase (JNK) signalling [58]. Grindelwald (Grnd), a receptor in this pathway [59], is expressed in the dorsal epidermis [60], but how the JNK pathway is activated in the DME cells remains unknown. Grnd may respond to signals from the determinants of apical cell polarity in the dorsal epidermis and/or to Egr, a ligand of Grnd, which is expressed in the amnioserosa. Egr transiently reduces tissue tension at the leading edge of the epidermis to allow transepithelial migration of macrophages at the extended germ band stage (stage 11) [61]. However, *egr* is not essential for germ band retraction and dorsal closure [42,62], whereas various other JNK pathway components are essential for this process [58,63]. Therefore, *egr* may not be essential for activating JNK signalling in the DME cells.

JNK activity promotes adhesion of the DME cells to the amnioserosa. This is achieved by increasing the contact zone between DME cells and peripheral amnioserosa cells (PAS cells) [19,64,65] and by forming a contractile actomyosin ‘cable’ at the leading edge of the dorsal epidermis [52,65,66]. These structural changes counter the pulling force that the amnioserosa exerts on the dorsal epidermis through different mechanisms including cell–matrix interactions, actomyosin contractility and cell volume decrease [64,66–73]. At the molecular level, JNK activity in the DME cells modulates the extracellular matrix [64,72–75] and promotes the expression of cytoskeletal and cell adhesion components [76–80]. This is achieved through a coherent feed-forward loop with the BMP gene *decapentaplegic (dpp)* [80,81]. Dpp signalling suppresses the transcription of *brinker* (*brk*), which encodes a repressor of JNK target genes in the DME cells, but Dpp also signals to the amnioserosa and lateral epidermis [52,65,82,83]. In particular, the PAS cells adopt their unique identity in response to JNK-Dpp activity in the DME cells and express elevated levels of *hnt* and *ush* during germ band retraction and dorsal closure [52,82]. Hnt antagonizes JNK signalling in the amnioserosa [58]. Ush functions in both the amnioserosa and the adjacent epidermis and promotes their adhesion [52]. In conclusion, JNK-dependent strengthening of adherence between the dorsal epidermis and amnioserosa and the pulling force of the amnioserosa provide the cellular basis for the morphogenetic roles of the amnioserosa in dorsal closure.

Seaming of the epidermis along the dorsal midline in the final phase of dorsal closure, also known as ‘zipping’ or ‘zippering,’ is initiated at the anterior and posterior canthi of the eye-shaped epidermal opening. Zipping is mediated by actin filaments and lamellipodia that ensure left and right cell matching and microtubule-dependent force generation [71,82,84,85]. Finally, anisotropic cell shape changes at the dorsal midline ‘efface’ the seam [86].

This leaves the question of how the amnioserosa drives germ band retraction. The amnioserosa folds over the most posterior end of the germ band and forms a so-called ‘dorsal bridge’. The bridge cells form lamellipodia that contact the underlying germ band and may drive the process of germ band retraction given that mutations in integrin and laminin genes do not form the dorsal bridge and interfere with germ band retraction [68,73]. Additionally, lateral amnioserosa cells exert a pulling force on the crook of the elongated germ band to advance germ band retraction [68,87,88].

## 5. Development and function of amnion and serosa after gastrulation in *Megaselia*

The serosa of *Megaselia* maintains *zen* expression when it spreads over the germ band [22]. Anterior, where no amnion cells are observed at this stage, the serosa slides directly over the head epidermis, while posterior and lateral, the leading edge of the serosa initially entrains the amnion, which folds back over the posterior germ band (forming a prominent dorsal bridge) and the lateral DME cells, before it dissociates from the amnion. Serosa spreading is facilitated by decoupling from the yolk sac and completed at the extended germ band stage (stage 10) roughly 2 h after its leading edge started to move freely about the germ band [26].

Given that germ band retraction and much of the dorsal closure process occur between serosa completion and serosa rupture, it is likely that both these morphogenetic movements are enabled by interactions between the amnion and the epidermis. *Megaselia* embryos subjected to Zen knockdown, which develop excess amniotic tissue and no serosa, proceed with germ band retraction and may even complete dorsal closure although the letter process is less robust [22] (see also [10,11]). Embryos subjected to knockdown of U-shaped group genes fail in germ band retraction and dorsal closure, presumably because of reduced amnion tissue [24,34].

During germ band retraction (stage 12), the amnion cells begin to extend cytoplasmic extensions over the yolk sac and redistribute to line the entire circumference of the epidermal opening by the time germ band retraction is completed (stage 13) (figure 3). The amnion cells do not contract during dorsal closure, but their cytoplasmic extensions contain F-actin and microtubules [25]. Injection of the microtubule-depolymerizing drug colcemid prevents amnion and epidermis closure, but degradation of injected colcemid in the amnion by ultraviolet light can rescue amnion closure [25]. The DME cells of *Megaselia*, like those of *Drosophila*, express *dpp* under the control of JNK signalling. Knockdown of JNK prevents the formation of an actomyosin cable at the boundary of epidermis and amnion and dorsal closure [25]. Taken together, these findings are consistent with a model in which JNK signalling in the DME cells of *Megaselia* secures the attachment of the epidermis to the amnion and that the amnion cells exert a microtubule-dependent pulling force on the leading edge of the epidermis that powers much of dorsal closure.

The dorsal opening in the amnion narrows over its entire length and forms a narrow slit by the time the serosa ruptures on the posterior-ventral side during stage 15 (figure 4*a–a*ʺ) [32]. The ruptured serosa contracts and accumulates within less than 30 min over the slit-like dorsal opening, where internalization of the serosa (figure 4*b–b*ʺ) is driven by actomyosin-dependent coordinated cell-shape changes of the serosa cells and by the microtubule-dependent seaming process of the amnion [25]. Accumulation and internalization of serosa cells remain incomplete and even revert when amnion closure (figure 4*c–c*ʺ) is experimentally prevented by microtubule depolymerization [25].

Whether the contracting serosa exerts a pulling force on the amnion has not been investigated in *Megaselia*. In species with an amniotic cavity, this seems to be the case [13–17]. However, in *Megaselia* the ruptured serosa rapidly slides over the amnion and the two tissues may not adhere tightly to each other. In any case, amnion closure requires removal of the contracted serosa, and the serosa cells seem to actively facilitate this process through their actomyosin-based contractile properties. The amnion does not form pronounced canthi and closes rather evenly along the entire length of the dorsal opening. However, dorsal closure of the epidermis over the amnion occurs via microtubule-dependent zipping at the anterior and posterior canthi, like in *Drosophila* [25].

## 6. Serosa and amnion features of the amnioserosa

The amnioserosa seems to reflect its dual origin because it combines developmental features of both the serosa and the amnion in *Megaselia*.

Serosa-like features of the amnioserosa include:

(i) *timing of specification*: serosa and amnioserosa cells are specified during blastoderm cellularization, whereas the amnion is specified at the beginning of gastrulation;
(ii) *requirement of* zen*:* BMP-dependent *zen* expression controls all aspects of serosa and amnioserosa specification. However, *zen* expression becomes independent of BMP-activity during gastrulation [24]. This change in *zen* regulation enables BMP-dependent amnion specification at the rim of the germ rudiment. It is unknown, whether a transition to BMP-independent *zen* expression also applies to gastrulating *Drosophila* embryos; and
(iii) *actomyosin-based contractility*: both serosa and amnioserosa cells contract during dorsal closure. In the amnioserosa, the PAS cells initiate contraction earlier than more central amnioserosa cells [69]. By contrast, the amnion cells have not contracted by the time the serosa is internalized and the amnion has closed [25]. Whether amnion cells contract during their internalization in the last phase of dorsal closure has not been examined.

Amnion-like features of the amnioserosa include:

(i) *morphogenetic roles in germ band retraction and dorsal closure*: both the amnioserosa and the amnion seem to play an active role in germ band retraction and dorsal closure. It is unlikely that the serosa has a function in germ band retraction because this morphogenetic movement occurs after serosa completion and before serosa rupture. For the same reason, an active role of the serosa in dorsal closure seems also unlikely apart from aiding its own internalization;
(ii) *pulling force on the leading edge:* the contact zone between epidermis and amnion or amnioserosa is reinforced through a similar genetic circuit that involves JNK-signalling and *dpp* expression in the DME cells and the formation of an actomyosin cable. However, the mechanisms of force generation seem to differ between the amnioserosa (actomyosin contractility and cell volume decrease) and the amnion (cytoplasmic extensions and microtubule-dependent force generation); and
(iii) *gene expression after germ band extension*: the amnioserosa of the extended *Drosophila* germ band shuts off *zen* expression and is then maintained under the influence of genes of the U-shaped group. Stage matched *Megaselia* embryos undergo serosa expansion and, while retaining *zen* expression in the serosa, restrict the expression of U-shaped-group genes, which are initially activated throughout the serosa and amnion, to the amnion. This is also the case for *egr*, which becomes a specific marker for amnion cells after germ band extension.

It should be noted that the amnion's role in morphogenesis is currently mostly inferred. Given that *M. abdita* is an excellent non-traditional experimental system for developmental genetic experiments, it should be possible to develop genetic tools to ablate the amnion in a stage-specific manner to assess its requirement in morphogenesis. Additionally, genetic tools to ablate the serosa prior to serosa expansion or prior to its rupture would help to assess whether the amnion functions autonomously in morphogenesis and whether dorsal closure is coupled to serosa rupture, as it is in some other species [13,14]. The comparison of the transcriptomes of amnion, serosa and amnioserosa cells during consecutive developmental stages by means of single-cell sequencing may also help to understand how amnion and serosa gene networks merged in the amnioserosa and could lead to new hypotheses about specific functions of these tissues.

## 7. Conclusion

The developmental trajectory of the amnioserosa combines cells, gene network components and functional properties of the serosa and amnion, as observed in *Megaselia*. Therefore, the amnioserosa provides a model for how tissues can merge in development and evolution. This obscure tissue, hardly known outside the community of insect embryologists, is therefore of conceptual interest in the field of evolutionary developmental biology. It will be worthwhile to explore the evolutionary history of this tissue in greater depths and to search for other examples where tissue-specific gene networks have been combined in evolution. Perhaps, merging tissue-specific developmental trajectories and functions is a more common evolutionary phenomenon than we currently like to believe.

## Data Availability

All data are included in the manuscript.
